# Disruption of *rimP-SC*, encoding a ribosome assembly cofactor, markedly enhances the production of several antibiotics in *Streptomyces coelicolor*

**DOI:** 10.1186/1475-2859-12-65

**Published:** 2013-07-02

**Authors:** Yuanyuan Pan, Cheng Lu, Hailing Dong, Lingjun Yu, Gang Liu, Huarong Tan

**Affiliations:** 1 Institute of Microbiology, Chinese Academy of Sciences, Beijing 100101, China

**Keywords:** rimP-SC, Streptomyces coelicolor, Actinorhodin, Calcium-dependent antibiotics

## Abstract

**Background:**

Ribosome assembly cofactor RimP is one of the auxiliary proteins required for maturation of the 30S subunit in *Escherichia coli*. Although RimP in protein synthesis is important, its role in secondary metabolites biosynthesis has not been reported so far. Considering the close relationship between protein synthesis and the production of secondary metabolites, the function of ribosome assembly cofactor RimP on antibiotics production was studied in *Streptomyces coelicolor* and *Streptomyces venezuelae*.

**Results:**

In this study, the *rimP* homologue *rimP-SC* was identified and cloned from *Streptomyces coelicolor*. Disruption of *rimP-SC* led to enhanced production of actinorhodin and calcium-dependent antibiotics by promoting the transcription of *act*II-ORF4 and *cdaR*. Further experiments demonstrated that MetK was one of the reasons for the increment of antibiotics production. In addition, *rimP-SC* disruption mutant could be used as a host to produce more peptidyl nucleoside antibiotics (polyoxin or nikkomycin) than the wild-type strain. Likewise, disruption of *rimP-SV* of *Streptomyces venezuelae* also significantly stimulated jadomycin production, suggesting that enhanced antibiotics production might be widespread in many other *Streptomyces* species.

**Conclusion:**

These results established an important relationship between ribosome assembly cofactor and secondary metabolites biosynthesis and provided an approach for yield improvement of secondary metabolites in *Streptomyces.*

## Introduction

In bacteria, more than 90% of energy is used in protein synthesis [1]. A large amount of them is used in ribosome assembly and protein translation. *In vitro* experiments have revealed that 50S and 30S ribosomal subunits could be reconstituted into active ribosomes from isolated components through heat-activation steps under different magnesium concentrations. However, these steps are not required and auxiliary proteins are needed *in vivo*[2]. An increasing number of ribosome assembly factors have been identified for 30S subunit reconstitution, such as RimP, RimM and RbfA in *Escherichia coli*[2].

RimP, formerly known as YhbC or P15a, is encoded by *rimP* in the *rbfA* operon and required for the maturation of 30S subunit. RimP is associated with 30S subunit but not 50S subunit or 70S ribosome. In the *rimP* deletion mutant, immature 16S rRNA is accumulated and the ribosomal profile shows fewer polysomes and the accumulation of unassociated 30S and 50S subunits. The difference becomes more obvious with the increasing temperature. The slow growth of *rimP* deletion mutant could not be suppressed by the increased expression of other known 30S maturation factors [2]. *In vitro* assembly studies showed that the preincubation of RimP with 16S rRNA could accelerate the binding rates of the 5′ domain ribosomal proteins S5 and S12 to almost all of the 3′ domain proteins (S3, S7, S9, S10, S13, and S14) [3,4].

*Streptomyces coelicolor* is the genetically most studied streptomycete and used as a model strain for studying the biology of actinomycetes [5,6]. It produces at least four distinct classes of antibiotics [6], including the well-known blue-pigmented aromatic polyketide antibiotic actinorhodin (ACT) which provides an easily tractable system for the methodological study of strain improvement [7], the red oligopyrrole prodiginine antibiotics (RED) [8], the acidic lipopeptide calcium-dependent antibiotics (CDA) [9] and methylenomycin [10]. The complete sequence and annotation of the *S. coelicolor* genome provide a way for its rational manipulation to identify potentially novel pathway products, and 29 predicted secondary metabolic gene clusters have been identified so far [11,12]. Besides screening new compounds, improving the production of existing compounds is still an important object, especially for the clinically and agriculturally applied antibiotics. Current main methods of improving antibiotics production include classical random mutation and ribosome engineering by the introduction of ribosomal protein mutations conferring drug resistance [13,14]. Although random mutation has played an important role in industry, its random nature is main drawback. In contrast, ribosome engineering approach allows for more rational manipulation.

In this paper, we cloned a *rimP* homologous gene *rimP-SC* from *S. coelicolor* and disruption of *rimP-SC* significantly increased the production of ACT and CDA. Meanwhile, the *rimP-SC* disruption mutant used as a heterologous expression host could produce more polyoxin or nikkomycin than the wild-type strain. In addition, disruption of *rimP-SV* also markedly improved jadomycin production in *Streptomyces venezuelae*, indicating that disruption of *rimP* homologues might be a widespread method for improving antibiotics production in *Streptomyces*.

## Results

### Identification of *rimP* homologue in *S. coelicolor*

**Figure 1:**
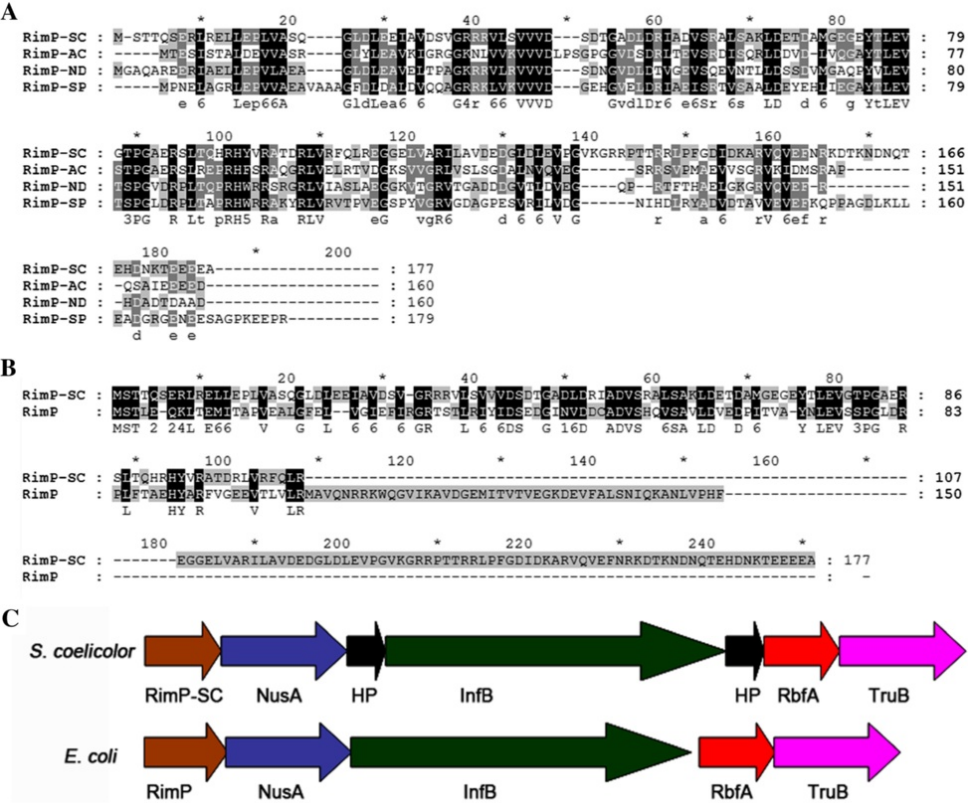
**Alignment of RimP from different bacteria and genetic organization of the *rimP-nusA-infB *operon. (A)** Alignment of RimP-SC with RimP-AC, RimP-ND and RimP-SP. **(B)** Alignment of RimP-SC with RimP. **(C)** Genetic organization of the *rimP-nusA-infB* operon in *S. coelicolor* and *E. coli*. RimP, ribosome maturation factor of *E. coli*; RimP-SC, RimP homologue of *S. coelicolor*; RimP-AC, RimP homologue of *Actinomyces* sp. *oral* taxon 848 str. F0332; RimP-ND, RimP homologue of *Nocardiopsis dassonvillei*; RimP-SP, RimP homologue of *Saccharomonospora paurometabolica*; NusA, the transcriptional elongation factor; InfB, the translational initiation factor; RbfA, ribosome binding factor; TruB, the tRNA (Ψ55) synthase; HP, hypothetical protein.

SCO5703 encodes a hypothetical protein consisting of 177 amino acids with a predicted molecular mass of 19.6 kDa. Comparative analysis demonstrated that its amino acid sequence was relatively conserved in actinomycetes (Figure 1A). However, its functional analysis has not been reported so far. Sequence alignment showed that it has 27% identity (41% similarity) with the RimP from *E. coli* (Figure 1B). Gene organization demonstrates that SCO5703 is flanked by genes similar to those found in *E. coli*, including the transcription elongation factor gene *nusA*, the translation initiation factor gene *infB*, the ribosome binding factor gene *rbfA* and the tRNA pseudouridine synthase gene *truB* (Figure 1C). The deduced product of SCO5703 contains the eukaryotic Sm or Sm-like (LSm) domain which associates with RNA to form the core domain of the ribonucleoprotein particle involved in a variety of RNA processing events including pre-mRNA splicing, telomere replication and mRNA degradation, making it a likely target for binding with the 30S ribosomal subunit in *S. coelicolor*.

To study the function of SCO5703, *E. coli rimP* disruption mutant (rimPDM) was constructed by PCR-targeting strategy. Then, the heterologous complemented strain of rimPDM (rimPDMC) was also constructed. Finally, the growth rates of *E. coli* wild-type strain BW25113, rimPDM and rimPDMC were detected at 28°C, 37°C or 42°C. As reported previously [2], rimPDM showed a reduced growth rate, especially at higher temperature. Introduction of the intact SCO5703 into the rimPDM restored the slow-growth phenotype almost to the wild-type level, indicating that SCO5703 is a functional homolog of *E. coli rimP* and thus is designated as *rimP-SC* (data not shown).

### Disruption of *rimP-SC* enhances antibiotics production in *Streptomyces*

**Figure 2:**
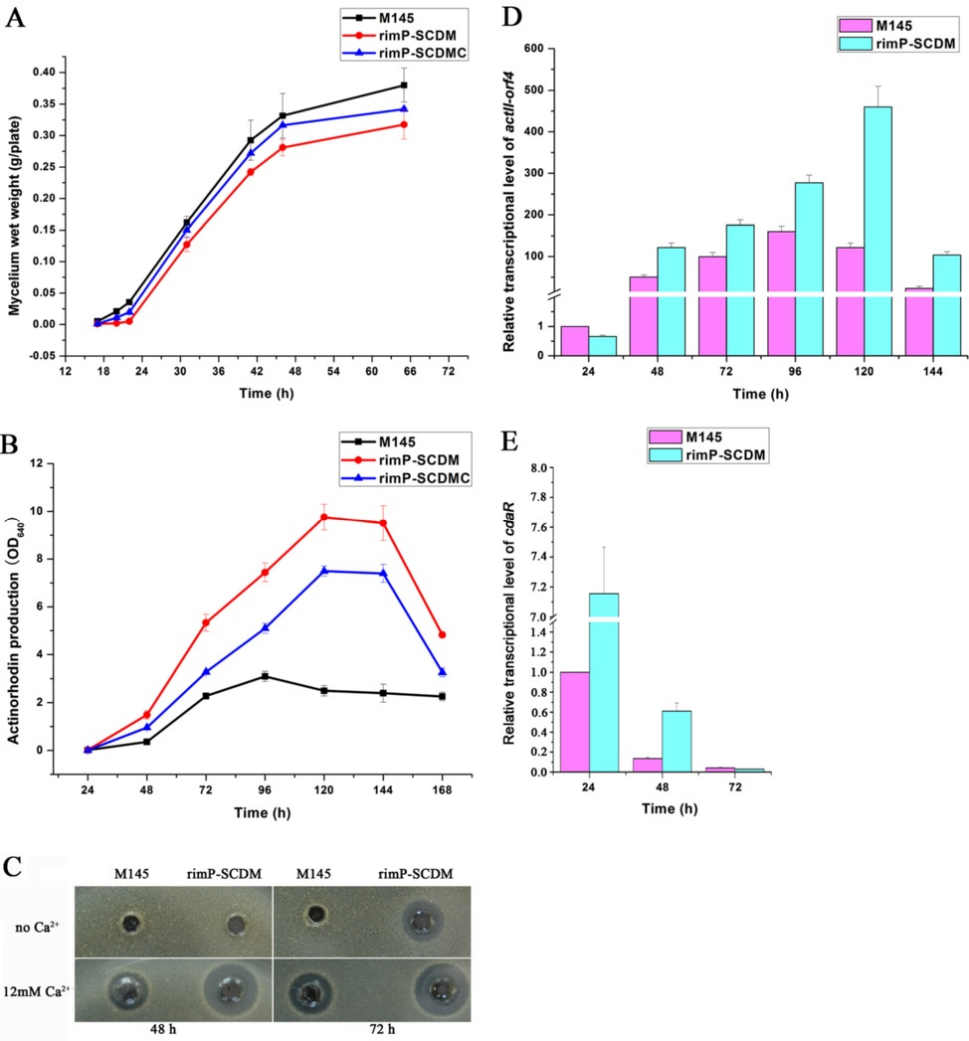
**Effects of *rimP-SC *disruption on biomass and production of actinorhodin and calcium-dependent antibiotics in *S. coelicolor*. (A)** Growth curve of *S. coelicolor* M145, rimP-SCDM and rimP-SCDMC. Biomass was calculated by mycelium wet weight in GYM plate. **(B)** Production of actinorhodin of *S. coelicolor* M145, rimP-SCDM and rimP-SCDMC in GYM medium. Cell cultures (50 mg) at each time point were treated with KOH (final concentration, 1 N) and the OD_640_ corresponding to 10 mg of mycelium was determined. **(C)** Bioassay of CDA in M145 and rimP-SCDM grown in DNA medium. **(D)** Transcriptional analysis of *act*II-ORF4 by real-time RT-PCR. The transcriptional level of *act*II-ORF4 was detected at 24, 48, 72, 96, 120 and 144 h in M145 and rimP-SCDM grown in GYM medium. **(E)** Transcriptional analysis of *cdaR* by real-time RT-PCR. The transcriptional level of *cdaR* was detected at 24, 48 and 72 h in M145 and rimP-SCDM grown in DNA medium. Data were presented as the averages of the results of three independent experiments in triplicate. Error bars showed standard deviations.

In order to clarify the function of *rimP-SC in vivo*, its disruption mutant (rimP-SCDM) was constructed via homologous recombination in *S. coelicolor* M145. Disruption of *rimP-SC* reduced the growth rate of *S. coelicolor* at initial period of rapid growth in GYM medium (Figure 2A). Interestingly, the ACT production of rimP-SCDM was remarkably increased 3-fold compared with the wild-type strain (Figure 2B). The growth rate and ACT production of the *rimP-SC* complemented strain (rimP-SCDMC) lay between the wild-type strain and rimP-SCDM (Figure 2A and B), suggesting that disruption of *rimP-SC* is the key reason for the enhancement of ACT production. Unlike ACT production, RED production of rimP-SCDM was similar to that of M145 in GYM medium (data not shown). Disruption of *rimP-SC* also enhanced CDA production in DNA medium (Figure 2C).

**Figure 3:**
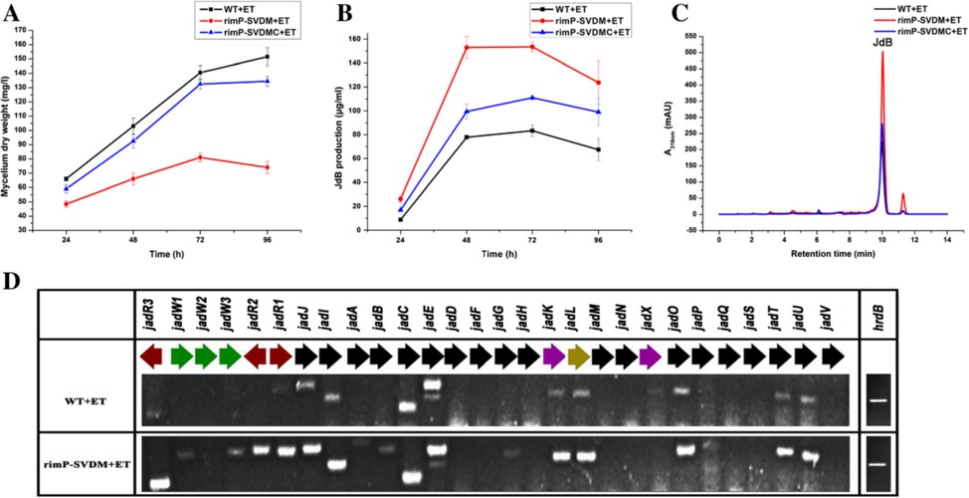
**Effects of *rimP-SV *disruption on biomass and jadomycin B production. (A)** Growth curve of the *S. venezuelae* wild-type strain (WT), rimP-SVDM and rimP-SVDMC with ethanol (ET) induction. Biomass was calculated by mycelium dry weight. **(B)** The yield of jadomycin B (JdB) in WT, rimP-SVDM and rimP-SVDMC. **(C)** HPLC analyses of fermentation filtrates from WT, rimP-SVDM and rimP-SVDMC grown for 48 h. mAU, milli absorbance units. **(D)** Transcriptional analysis of jadomycin B biosynthetic gene cluster by RT-PCR. Total mycelial RNA extracted after 24 h fermentation was used as template.

To examine the function of *rimP* homologues in other streptomycetes, the *rimP* homologous gene *rimP-SV* was disrupted in *S. venezuelae*. As in *S. coelicolor*, disruption of *rimP-SV* remarkably reduced the growth rate of *S. venezuelae* in liquid MYM medium. And the growth rate of complemented strain (rimP-SVDMC) was almost restored to the level of the wild-type strain (Figure 3A). HPLC analysis showed that the production of jadomycin B had 2–3 folds increase in the *rimP-SV* disruption mutant (rimP-SVDM) (Figure 3B and C). Meanwhile, the jadomycin B production in the complemented strain was almost restored to the wild-type level, albeit slightly higher than the wild-type strain (Figure 3B and C). The disruption and complementation experiments suggested that *rimP-SV* was a key determinant in jadomycin B biosynthesis. Transcriptional analysis further confirmed that the increment of jadomycin production was due to the increased transcription of jadomycin biosynthetic genes (Figure 3D). Thus, the stimulatory effect on antibiotics production owing to the disruption of *rimP* homologues is not confined to one *Streptomyces* species.

### Transcriptional analysis of *act*II-ORF4, *redD* and *cdaR*

To explain the reasons for the enhanced production of several distinct antibiotics in rimP-SCDM, the transcription of corresponding biosynthetic genes were measured by real-time RT-PCR. The transcriptional levels of their pathway specific regulatory genes (*act*II-ORF4, *redD*, *cdaR*) involved in the biosynthesis of three well-known antibiotics (ACT, RED and CDA) were determined in M145 and rimP-SCDM. The transcription of *act*II-ORF4 reached the highest level in rimP-SCDM at 120 h and was 3-fold higher than that in M145 (Figure 2D). Consistent with ACT production and transcription of *act*II-ORF4, the transcriptional levels of SCO5072, SCO5082, SCO5086 and SCO5087 involved in ACT biosynthesis were also increased in rimP-SCDM (data not shown). Consistent with RED production, the transcriptional level of *redD* had no significant difference between rimP-SCDM and M145 (data not shown). The transcriptional level of *cdaR* in rimP-SCDM exceeded 6-fold more than that in M145 at 24 h, and the difference was narrowed from 72 h to 120 h (Figure 2E). Through the transcriptional analysis of the pathway-specific regulatory genes *act*II-ORF4/*cdaR* and biosynthetic genes involved in the ACT/CDA production, we might conclude that *rimP-SC* affects the ACT/CDA biosynthesis by controlling the transcription of pathway-specific regulatory gene *act*II-ORF4/*cdaR* and ACT/CDA biosynthetic genes.

### RimP affects the translational efficiency and fidelity in *E. coli*

**Figure 4:**
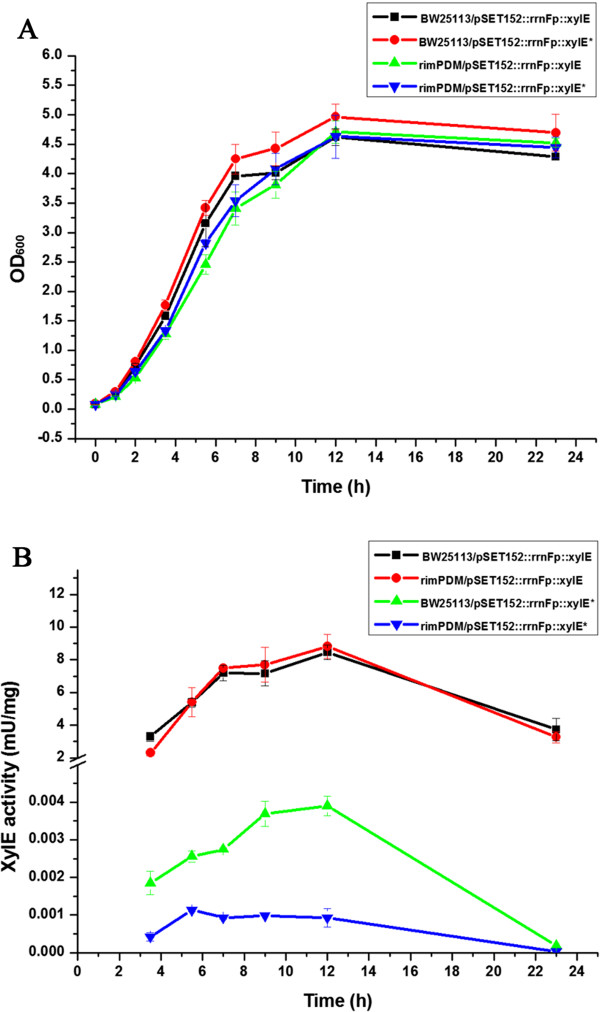
**Activity analysis of catechol dioxygenase in *E. coli*. (A)** Growth curve of four different *E. coli* strains. **(B)** Activity analysis of the wild-type or mutated catechol dioxygenase. Data were presented as the averages of the results of three independent experiments in triplicate. Error bars showed standard deviations. The asterisk (*) indicates mutated catechol dioxygenase (UGG codon as tryptophan at 47 position was replaced by UGA as stop codon).

As a ribosome assembly cofactor, RimP may affect translational efficiency. Thus, the translational accuracy was measured using the mutated *xylE* as a reporter which contains a UGA stop codon instead of a UGG tryptophan codon at 47 position. When the stop codon was decoded by a near-cognate tRNA, the full length catechol dioxygenase was expressed and showed enzyme activity. To check the expression of catechol dioxygenase, all recombinant strains (BW25113/pSET152::rrnFp::xylE, rimPDM/pSET152::rrnFp::xylE, BW25113/pSET152::rrnFp::xylE^*^ and rimPDM/pSET152::rrnFp::xylE^*^) were cultured in LB medium at 37°C. Under this condition, the growth rate of BW25113 was a little faster than rimPDM (Figure 4A). Meanwhile, the expression level of wild-type catechol dioxygenase in BW25113 was almost the same as rimPDM (Figure 4B). When introducing a UGA stop codon into the wild-type *xylE* (named as xylE^*^), the catechol dioxygenase activity decreased 3 orders of magnitude. Meanwhile, the activity of XylE^*^ decreased almost 2–4 folds in rimPDM compared with BW25113 (Figure 4B). As shown in Figure 4B, the translational error rate of mutated catechol dioxygenase was about 5 × 10^-4^ in BW25113 and 1-2 × 10^-4^ in rimPDM. Therefore, the presence of RimP remarkably increased misreading of the UGA stop codon probably by decoding the near-cognate tRNA. These results implied that the presence of RimP might facilitate misreading of codons to result in the fast growth during exponential phase, but decrease the translational accuracy.

### Disruption of *rimP-SC* significantly enhanced the expression of MetK

**Figure 5:**
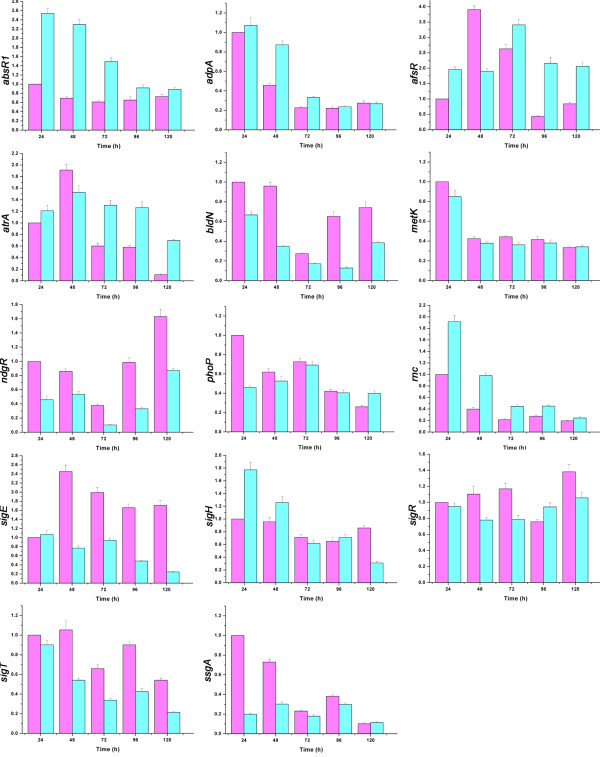
**Transcriptional analysis of genes encoding global activators and repressors as well as sigma factors by real-time RT-PCR.** The transcriptional levels were detected after fermentation for 24, 48, 72, 96 and 120 h in GYM medium. Data were presented as the averages of the results of three independent experiments in triplicate. Error bars showed standard deviations.

**Figure 6:**
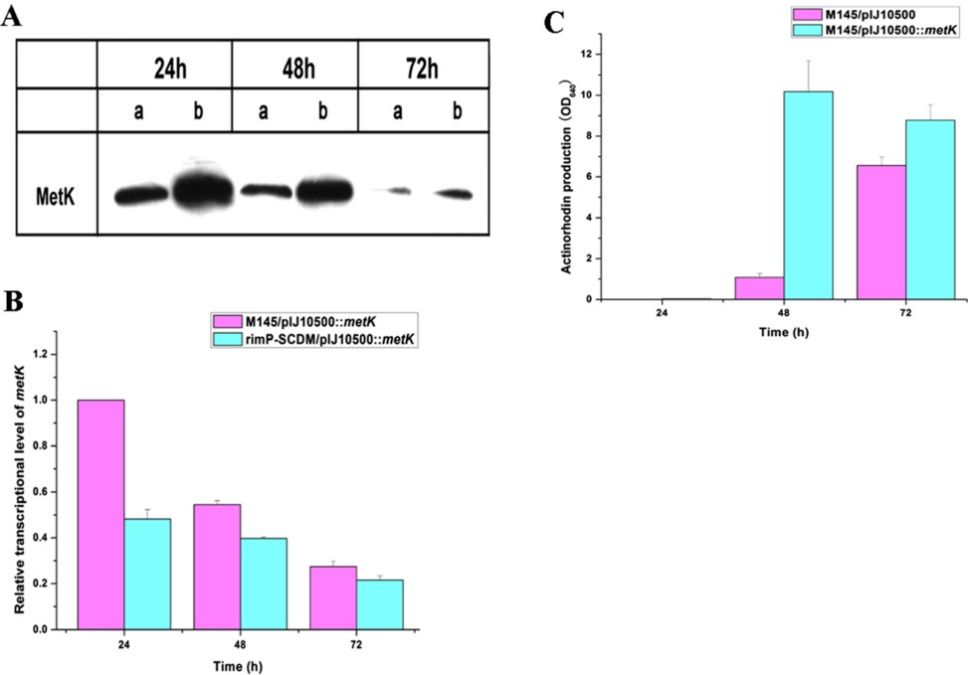
**Effect of *rimP-SC *disruption on MetK expression. (A)** Detection of MetK by western blotting analysis. **(B)** Transcriptional analysis of *metK* by real-time RT-PCR. **(C)** The production of actinorhodin. Cell cultures (50 mg) at each time point were treated with KOH (final concentration, 1 N) and the OD_640_ corresponding to 10 mg of mycelium was determined. Data were presented as the averages of the results of three independent experiments in triplicate. Error bars showed standard deviations.

To study how disruption of *rimP-SC* enhance ACT/CDA production in *S. coelicolor*, transcriptions of six global activator genes (*absR1, adpA, afsR, atrA, metK* and *rnc*) and three global repressor genes (*phoP, ndgR* and *ssgA*) as well as five sigma factor genes (*bldN, sigE, sigH, sigR* and *sigT*) were analyzed by real-time RT-PCR. The results indicated that the transcriptions of five activator genes (*absR1, adpA, afsR, atrA* and *rnc*) were increased and the transcriptions of three repressor genes (*phoP, ndgR* and *ssgA*) were decreased in rimP-SCDM (Figure 5). Although the transcriptional changes of these genes could explain the increase of ACT production, it was unclear whether *rimP-SC* affected antibiotics biosynthesis at the translational level. Therefore, *metK* and *sigR*, whose transcription was not changed significantly in comparison with the wild-type strain, were selected for further studying their translations. To check the syntheses of MetK and SigR, the flag-tagged system was used. The result of western blotting showed that the expression of MetK was much stronger in rimP-SCDM than M145 from 24 h to 72 h (Figure 6A). However, the transcription of *metK* in rimP-SCDM did not exceed M145 (Figure 6B). In addition, the expression of SigR did not show significant difference between the wild-type strain and rimP-SCDM (data not show). In agreement with previous report [15], our results also showed that over-expression of MetK obviously stimulated ACT production in advance and led to an increase of ACT production up to 140% compared with the control strain M145/pIJ10500 (Figure 6C). Therefore, disruption of *rimP-SC* increased translational level of proteins related to secondary metabolites, such as MetK which may be one example of many proteins. The complicated mechanism that disruption of *rimP* homologues led to enhanced production of antibiotics is intriguing, but remains unclear, so it is worthy of studying and is being explored deeply in our lab at present.

### RimP-SCDM improved the production of polyoxin and nikkomycin

**Figure 7:**
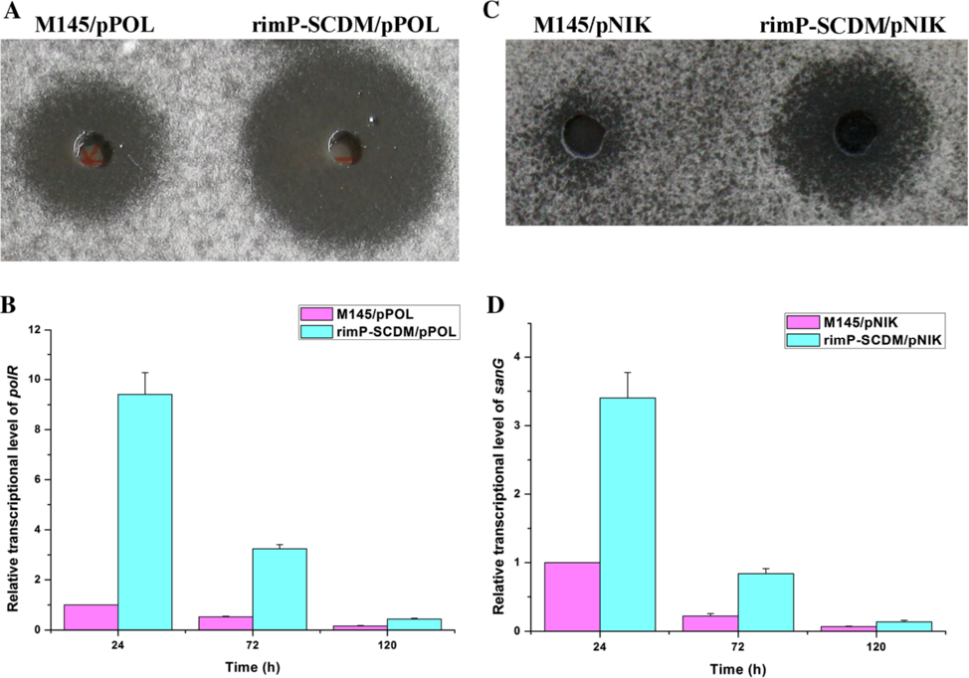
**Heterologous expression and transcriptional analysis of polyoxin and nikkomycin biosynthetic gene clusters. (A)** Bioassay of polyoxin. **(B)** Transcriptional analysis of *polR* by real-time RT-PCR. **(C)** Bioassay of nikkomycin. **(D)** Transcriptional analysis of *sanG* by real-time RT-PCR. Data were presented as the averages of the results of three independent experiments in triplicate. Error bars showed standard deviations.

Recombinant strains containing the entire polyoxin or nikkomycin biosynthetic gene cluster were cultured for 5 days and their fermentation broths were measured by bioassay. The results showed that the fermentation broth of rimP-SCDM/pPOL had obvious stronger bioactivity than that of M145/pPOL against the indicator strain *A. longipes* (Figure 7A). Meanwhile, the rimP-SCDM/pPOL and M145/pPOL had comparable growth rates and final biomass (data not shown). Subsequently, we checked the transcription of *polR* involved in the biosynthesis of polyoxin. The results showed that the transcription of *polR* in rimP-SCDM/pPOL was 8-fold higher than that in M145/pPOL at 24 h (Figure 7B), which were consistent with the results of bioassay. Meanwhile, the fermentation broth of rimP-SCDM/pNIK had obvious stronger bioactivity than that of M145/pNIK against the indicator strain *A. longipes* (Figure 7C). The transcription of *sanG* encoding a positive regulator for nikkomycin biosynthesis was measured. As expected, the transcription of *sanG* in the rimP-SCDM/pNIK was increased 2.5-fold compared with M145/pNIK at 24 h (Figure 7D). Above results showed that rimP-SCDM could significantly improve the yield of polyoxin and nikkomycin. Therefore, it is possible that rimP-SCDM can be used as the promising heterologous expression host.

## Discussion

SCO5703 is the homologue of RimP which facilitates the maturation of the 30S subunit in *E. coli*. Since RimP affects the formation of polysomes in *E. coli*[2], it is possible that disruption of *rimP-SC* also reduces the formation of polysomes and leads to the production of 30S subunit containing immature 16S rRNA in *S. coelicolor*. In addition, RimP-SC was involved in the antibiotics production in *S. coelicolor*. We postulate that the difference in the growth rate and antibiotics production correlate with the amount of polysomes which affects protein translational capacity.

Ribosomal protein S12, which is located at the interface of 30S and 50S subunits and closes to the decoding center of the ribosome, is important in maintaining translational accuracy. Contact of S12 and 16S rRNA facilitates the formation of closed conformation of 30S ribosomal subunit and hampers the entrance of near-cognate tRNA during translation [16]. The closed conformation activates EF-Tu, GTPase and ribosomes to enter the translational process [17,18]. Aside from drug resistance, many S12 mutant strains show pleiotropic effects including translational hyper-accuracy, reduced growth and impaired peptide chain elongation [19]. The K88E mutation of the S12 protein causes a high level of resistance of *S. coelicolor* to streptomycin and stimulates the production of ACT [20]. These phenomena might be due to the increased protein synthesis during the late growth phase and the enrichment of ribosome recycling factor RRF [21]. The phenomenon that disruption of *rimP-SC* increases protein synthesis at the late growth phase is similar to the K88E mutant of S12 protein [22,23]. Unlike the K88E mutant, deletion of *rimP-SC* may not increase the stability of 70S ribosome as the *rimP* mutant only hindered the maturation of 30S subunit and did not result in the change of S12 protein in *E. coli*[2]. In addition, accumulation of ppGpp stimulates antibiotics production in *S. coelicolor*[24]. The increased production of ACT in S12 mutant results from higher level of ppGpp [25]. However, the amount of ppGpp had no obvious difference between M145 and rimP-SCDM in our study (data not shown), indicating that ppGpp was not the reason for improved production of ACT in RimP-SCDM. The similar phenomenon that the hyperaccurate ribosomes exhibited slightly reduced rates of GTP hydrolysis for both cognate and near-cognate ternary complexes has been reported [26]. Therefore, the reasons for stimulating antibiotics production due to *rimP-SC* disruption are different from the mutation of S12 protein in *S. coelicolor*.

The onset of protein synthesis is determined by tRNA selection. Generally, the tRNA selection is divided into an initial selection and a later proofreading process. During initial selection, cognate aminoacyl-tRNA facilitates the stabilization of a closed 30S conformation. However, near-cognate aminoacyl-tRNA, which differs from cognate tRNA by a single, subtle mismatch in codon-anticodon base-pairing and cannot be accurately distinguished on the basis of difference in the free energy of base-pairing alone, is not disadvantage for the stabilization of a closed 30S conformation [17]. Stabilization of the closed form of 30S ribosomal subunit could reduce the translational fidelity and increase the translational speed. The translational accuracy was measured using *xylE* gene as a reporter in *E. coli* wild-type strain and rimPDM. The results showed that RimP might stabilize the closed form and accelerate the reconstitution of 30S ribosomal subunit induced by cognate or near-cognate tRNA, thus speeding up the translation. Without RimP, the closed 30S form might be unstable and unfavorable for selection of near-cognate tRNA, thus leading to higher translation fidelity and lower translation speed.

## Conclusions

As in *E. coli*, RimP-SC encoded a cofactor involved in ribosome assembly of the 30S subunit and its disruption reduced growth rate at initial period of rapid growth in *S. coelicolor*. RimP-SC also played an essential role in actinorhodin and calcium-dependent antibiotics production. Disruption of *rimP-SC* enhanced expression of MetK and protein translational accuracy, resulting in increased antibiotics production. This is the first study to address the relationship between ribosome assembly cofactor and antibiotics production. Our results provided an approach for yield improvement based on *rimP* homologues disruption, which was also effective in *S. venezuelae*, implying that the approach might be adopted to increase antibiotics production in other *Streptomyces* species. Ultimately, more peptidyl nucleoside antibiotics—polyoxin and nikkomycin could be produced in *rimP-SC* disruption mutant than M145, indicating that *rimP-SC* disruption mutant could be used as a promising host for heterologous expression.

## Materials and methods

### Bacterial strains, plasmids, primers, growth conditions and assay of antibiotics

Bacterial strains and plasmids used in this study were listed in Table 1. The primers used in this study were listed in Table 2. *E. coli* JM109 was used as a general host for routine cloning experiment. BW25113 was used as an *E. coli* host for the construction of *rimP* disruption mutant via λ-Red-mediated recombination technology. *E. coli* ET12567/pUZ8002 was used as a host for transferring DNA from *E. coli* to *Streptomyces* by intergeneric conjugation. *S. coelicolor* M145 was a derivative of the wild-type strain *S. coelicolor* A3(2) lacking plasmids SCP1 and SCP2. *S. venezuelae* ATCC10712 was the wild-type strain of *S. venezuelae*. *Alternaria longipes* was the indicator strain of polyoxin and nikkomycin. *Staphylococcus aureus* was the indicator strain of calcium-dependent antibiotics (CDA). Usually, *E. coli* and its derivatives were grown at 37°C. *Streptomyces* and their derivatives were grown at 28°C. Yeast extract-malt extract liquid medium (YEME), GYM medium, Difco Nutrient Agar (DNA) medium supplemented with 0.5% NaCl (w/v) and agar minimal medium (MM) supplemented with mannitol as sole carbon source were prepared for growth and sporulation of *Streptomyces* as described previously [6]. SP medium (3% mannitol, 1% soluble starch, 0.75% yeast extract, and 0.5% soy peptone, pH 6.0) for polyoxin and nikkomycin production was prepared as described previously [27]. Potato dextrose agar medium (PDA) for the detection of polyoxin and nikkomycin was used. MYM medium for jadomycin production was prepared as described previously [28]. For the production of ACT and RED, fresh spores of *S. coelicolor* were inoculated in GYM medium as described previously [6]. ACT and RED were detected spectrophotometrically [6]. CDA production in DNA medium was assayed as described previously [29]. Jadomycin production was measured as described previously [28,30,31]. For fermentation, spore suspensions of *S. venezuelae* were inoculated in MYM and grown at 28°C on a rotary shaker (220 rpm) for 24 h as seed cultures. 5 ml (5% v/v) of seed culture was transferred into flasks containing 100 ml of galactose-isoleucine medium with 1.5 ml ethanol, and then cultured at 28°C on a rotary shaker (220 rpm) for jadomycin production. Jadomycin B was identified by HPLC analysis (Agilent 1260 HPLC and RPC-18) at 316 nm absorption wavelengths. Chemical reagent, mobile phase and gradient elution process were performed as described previously [31].

**Table 1:** Bacterial strains and plasmids used in this study

**Bacterial strains/plasmids**	**Relevant characteristics**	**Source/references**
*Streptomyces*		
*S. coelicolor* M145	A derivative of the wild-type strain *S. coelicolor* A3(2) lacking plasmids SCP1 and SCP2	[6]
rimP-SCDM	The *rimP-SC* disruption mutant of *S. coelicolor*	This study
rimP-SCDMC	The complemented strain of rimP-SCDM	This study
M145/pNIK	*S. coelicolor* M145 containing the entire nikkomycin biosynthetic gene cluster	This study
rimP-SCDM/pNIK	rimP-SCDM containing the entire nikkomycin biosynthetic gene cluster	This study
M145/pPOL	*S. coelicolor* M145 containing the entire polyoxin biosynthetic gene cluster	This study
rimP-SCDM/pPOL	rimP-SCDM containing the entire polyoxin biosynthetic gene cluster	This study
*S. venezuelae* ATCC10712	The wild-type strain of *S. venezuelae*	[6]
rimP-SVDM	The *rimP-SV* disruption mutant of *S. venezuelae*	This study
rimP-SVDMC	The complemented strain of rimP-SVDM	This study
*E. coli*		
JM109	*recA1*, *endA1*, *gyrA96*, *thi-1*, *hsdR17*, *supE44*, *relA1*, Δ(lac-proAB)/F’[traD36, proAB+, lacIq, lacZΔM15]	Invitrogen
BW25113	K-12 derivative; ΔaraBAD ΔrhaBAD	[32]
ET12567/pUZ8002	*dam dcm hsdS cat tet*/pUZ8002	[33]
rimPDM	The *rimP* disruption mutant of *E. coli* BW25113	This study
rimPDMC	The heterologous complemented strain of rimPDM	This study
rimPDM/pSET152::rrnFp::SCO5703	rimPDM containing pSET152::rrnFp::SCO5703 for heterologous complementation analysis	This study
BW25113/pSET152::rrnFp::xylE	BW25113 containing pSET152::rrnFp::xylE for catechol dioxygenase assay	This study
BW25113/pSET152::rrnFp::xylE*	BW25113 containing pSET152::rrnFp::xylE* for catechol dioxygenase assay	This study
rimPDM/pSET152::rrnFp::xylE	rimPDM containing pSET152::rrnFp::xylE for catechol dioxygenase assay	This study
rimPDM/pSET152::rrnFp::xylE*	rimPDM containing pSET152::rrnFp::xylE* for catechol dioxygenase assay	This study
Plasmids		
pRIMPSC3	Plasmid used for the construction of rimP-SCDM	This study
pRIMPSC4	pSET152 containing the intact *rimP-SC* with its putative promoter	This study
pRIMPSV3	Plasmid used for the construction of rimP-SVDM	This study
pRIMPSV4	pSET152 containing the intact *rimP-SV* with its putative promoter	This study
pSET152::rrnFp::xylE	pSET152 containing the wild-type *xylE* and the promoter of *rrnF* for activity detection of catechol dioxygenase	This study
pSET152::rrnFp::xylE*	pSET152 containing the mutated *xylE* and the promoter of *rrnF* for activity detection of catechol dioxygenase	This study
pIJ10500::*metK*	pIJ10500 containing the intact *metK* with its promoter	This study
pIJ10500::*sigR*	pIJ10500 containing the intact *sigR* with its promoter	This study
pNIK	pSET152 containing the entire nikkomycin biosynthetic gene cluster	[34]
pPOL	pSET152 containing the entire polyoxin biosynthetic gene cluster	[35]

**Table 2:** Primers used in this study

**Genes and primers**	**Sequence (5′-3′)**
*rimP* homologues relevant primers	
Lrimp-SC-F	CCCAAGCTTGGCCAGCCGGTCCTCCAGTT
Lrimp-SC-R	GCTCTAGAGGTGGTGCTCATCCGGGTGA
Rrimp-SC-F	GGGGTACCAGGCCCACCACCCGCAGACT
Rrimp-SC-R	GGAATTCCGGCGTGCGGCTTGGATCTA
CrimP-SC-F	CGCGGCCAGTTCCTCACTGT
CrimP-SC-R	CAGGGCGCTCATGTCGATGT
ECrimP-F	TTGTCCACATTAGAGCAAAAATTAACAGAGATGATTACTGGAATTGTGAGCGGATAAC
ECrimP-R	TCTGGATATTACTCAGCGCGAACACTTCATCTTTACCTTAGGCGATTAAGTTGGGTAA
YZECrimP-F	TTGTCCACATTAGAGCAAA
YZECrimP-R	TTAAAAGTGGGGAACCAG
PFrimP-F	CGGGATCCGACCCACAACAGCACACG
PFrimP-R	GCTCTAGATCTCCTTCTCCCGTACCAA
LrimP-SV-F	AAGCTTGCCGAACGGTACAGAAAGGGTA
LrimP-SV-R	TCTAGATCGCTCTGGGTGGTGCTCATC
RrimP-SV-F	GGATCCGGCGAGTACGTCCTCGAAGT
RrimP-SV-R	GATATCACCTTGCTCTCCACACCGAACTCC
CrimP-SV-F	CGGCGGTTCGAAACCCATGC
CrimP-SV-R	CTACGCCTCCTCTTCCTTCTTGTCCTT
YZrimP-SV-F	GTGGTACGTCGTCGAAGATC
YZrimP-SV-R	GCAAAGCGTCAGTCAACTTG
Primers for real-time PCR of genes in *S. coelicolor*	
RTact-F	GCTCCTCAGGCGGCACGA
RTact-R	GCCGGCGGGTGTGGTACA
RTred-F	GCCCTGACGCGCTATTGG
RTred-R	GGTGGTGGGCGAGACGGA
RTcda-F	GGAAAGCGACGCCTACTT
RTcda-R	AGGCTCGTCTTTCCGATT
RTmetK-F	CGAGCCCGTGGGTCTGTT
RTmetK-R	CAGGTCGAGAGCGCGGAT
RTsigR-F	CGACCACCTGCCCGACTC
RTsigR-R	CCCCATGATGTCCGCGAT
RTsigE-F	GGAGGAGGTGCCGACCGA
RTsigE-R	TTCCCGCCGACATTCCGA
RTsigH-F	GGAGCCGCTGGACGACCT
RTsigH-R	CACCGCCCAGCCCTTGTC
RTbldN-F	GACAGCGCCCGCATGATG
RTbldN-R	GAGCGCCCGCAGAAAGGT
RTsigT-F	GCCCTCGTCTCCGCCTAC
RTsigT-R	CAGGCGTTCGGTGTCGTC
RTabsR1-F	CCCGCAGTCGATCATGGA
RTabsR1-R	GCAGGGCGAACTCCTTGTC
RTadpA-F	AGCACCTCCACGAGCAGTTC
RTadpA-R	CGTCCACCGAGTAGTCCGA
RTafsR-F	GGCGGTGGATCTGCTGTG
RTafsR-R	ACATCGCTGAGAACGGTGC
RTatrA-F	CCGGCGGTGCGATGAGTA
RTatrA-R	ACCCCAGCTCGCCGAACA
RTndgR-F	CGACGTGACGGGCGAGAG
RTndgR-R	GGAGCCGGCCTTCATGGT
RTphoP-F	ACGTTCCCGTGATCATGGTG
RTphoP-R	CAGTACGGCTCGGATGCG
RTrnc-F	GGTGATCGGCGCGGTCTA
RTrnc-R	CCTTCGGTCGCGGTGAGT
RTssgA-F	CAGGCGCTGTTCCGTTCC
RTssgA-R	GATGCGGTCCAGGGCCTC
RT5072-F	GACGACCTGCCGCTCAAG
RT5072-R	GAACGATGTGCGGTGGGT
RT5082-F	GGAGGCCCTGGAGCAGTC
RT5082-R	GCCGGCGATGATGATCTC
RT5086-F	ACCTCACCGGCGTGTTCC
RT5086-R	CGTGCTTCGAGGCGGAGT
RT5087-F	GAACGACCGCCACGAGAC
RT5087-R	GATCTCCAGCGAGCCGAT
RTsanG-F	GGCCACCCTGCAGACGTAC
RTsanG-R	CGGGACAGGTCGAACGTG
RTpolR-F	GGTCTCCCGCGGACAACA
RTpolR-R	GCGGCTCGTAGGACGTGA
RThrdB-SC-F	GATCGCCGAGTCCGTCTC
RThrdB-SC-R	CACTGAGTGGCCGGAATC
Primers for real-time PCR of genes in *S. venezuelae*	
RTjadR3-F	CACGTGGACGTGACGGATACGG
RTjadR3-R	GGGTGTCGGCGAGGTTTCCTTC
RTjadW1-F	TCGTCTGCTCCGACATCACCC
RTjadW1-R	GCAGGAAGGAGACGCTCAGGTC
RTjadW2-F	ACGTACTGATCCACTGCGCCTCC
RTjadW2-R	CGATCAGGGAGTGCAGCGAGG
RTjadW3-F	ACTACGGCAGCAACGAGAAGGC
RTjadW3-R	AGGGCGAGGGTCATCGTGTC
RTjadR2-F	TCGGCGATCAGTTCGGGAGC
RTjadR2-R	AGCCATTCGCCGTTGTCCC
RTjadR1-F	TGGACGGCTTGGAGGTCTGC
RTjadR1-R	GGCTGCTCACATGGGTGTCG
RTjadJ-F	CTGTCGGAGGCTCAGAACGC
RTjadJ-R	ACGATCACGTTCGCAAGCAG
RTjadI-F	TGCACAGCACTCTGATCGTGG
RTjadI-R	GCGTTCGCCTCCCAGTTGTAG
RTjadA-F	CCCCAACACCGTGGTCTCC
RTjadA-R	GCGGTCGTTCTGCTTGGTG
RTjadB-F	GGAGCCAGGGCAGCCAGTAC
RTjadB-R	CGAAGGTGGAGCCGTATCCG
RTjadC-F	GCAGCAAGACCTTCACCCTCG
RTjadC-R	CCGACAGGTGCGCGTTGAC
RTjadE-F	GCCGACGAGCTGTGGAACG
RTjadE-R	GAGGCCAGGTAGCCGACGAG
RTjadD-F	GCCTTGCTGCACGACTACCG
RTjadD-R	ACGCCGTCCTCGTTCTCCTC
RTjadF-F	ACGCCGCTCTGGGTGAACT
RTjadF-R	GATGTCGAGTCCTGAGACCTTGC
RTjadG-F	ACCTGACCGTCTTCAACCTCTTCG
RTjadG-R	TGCTGCTGGTCCGGCTTCAC
RTjadH-F	GACGACGACGCCGTGGAGA
RTjadH-R	GATGTCCTCGCCCGTGATGC
RTjadK-F	CGGCTGCGGACAGGAGTACG
RTjadK-R	GAGGCCCAGGCTGATGTTGTG
RTjadL-F	GGAAGGAGGAACGGAAGGACG
RTjadL-R	ATCAGGGTGTAGAGGGCGAGG
RTjadM-F	CCCGCTACACCGGAGTCCC
RTjadM-R	GAGTCCCGTGCCGAGTCCC
RTjadN-F	GCAGGGTTTCGGTCTGGAGG
RTjadN-R	CGAGGCCGTTCTGGGTGATC
RTjadX-F	CCACCACCGACCTCACCG
RTjadX-R	CGAAGTGGGCGGAGGGC
RTjadO-F	TTCCACAAGTCCAACCGCAAC
RTjadO-R	TTCGATCAGCGGCTGGGTC
RTjadP-F	AAGCACGTCCTGGCCGAGAAG
RTjadP-R	GGTCCATGCCGAAGGCGATGT
RTjadQ-F	CGACAAGCCGATGATCTACTACCC
RTjadQ-R	GCGTGAGGTTCTTGGCGATGT
RTjadS-F	GTCTTCCCGCCCAACCACG
RTjadS-R	GCGAGGGAGCCAGCGTCAC
RTjadT-F	CGACGAGGTGTACGGCACG
RTjadT-R	CAGGATGCGTTCGGTCAGG
RTjadU-F	CAGGTCAATCAGGTCAGCCACA
RTjadU-R	CCGGTCGGACAGGATCAGC
RTjadV-F	GACGAGCCGCAGGGCGAG
RTjadV-R	CCGCTCCGCCACCATCCG
RThrdB-SV-F	AGATTCCGCCAACCCAGTG
RThrdB-SV-R	GAGCGTCGTCTCGTCTCGTC
other primers	
rrnFp-F	TGGAGGGAGATACGAGAACG
rrnFp-R	CCCAGAGTGAAGGGCAGATT
MxylE-F	TGAACCGAAGTGGATAAGTT
MxylE-R	AGCCTTCAGATAGACACGGC
metK-F	CGGCGGCTGGAATGAATGACCC
metK-R	CAGGCCCGCGGCCTCGCGCA
sigR-F	GGGCGGAGATCAGCCAGGAAAG
sigR-R	TGACCCCGAGCCTTTCGCTTCGT
polB-F	GGTGAAGACGCCAACGAC
polB-R	GATCGGAGCGCGTACCAG
sanG-F	GGGGTACCGTGCGTCAACCTCATCCCG
sanG-R	GGAATTCGCTTGCCCGCTGGTCT
Kan-F	TCTAGAGATCCCCTGGATACCGCTCG
Kan-R	GGATCCGTACCCGAACCCCAGAGTC

Plasmids pBluescript KS+, pEASY-Blunt and pUC119::kan were used for routine cloning experiments in *E. coli*. The *Streptomyces—E. coli* shuttle plasmid pKC1132 was used to construct gene disruption mutants via homologous recombination. The integrative plasmid pSET152 was used to introduce a single copy of *rimP* homologue gene into the *Streptomyces* chromosome. pIJ10500 containing hygromycin B (*hyg*) resistance gene and 3 × FLAG tag was used in western blotting experiment. The *xylE* from pIJ4083 was used for the construction of the reporter system. When necessary, antibiotics were used at the following concentrations: ampicillin (100 μg · ml^−1^), kanamycin (100 μg · ml^−1^), apramycin (100 μg · ml^−1^) or hygromycin B (50 μg · ml^−1^) in LB for *E. coli*; Nalidixic acid (25 μg · ml^−1^), apramycin (100 μg · ml^−1^) or kanamycin (100 μg · ml^−1^) in MS for *Streptomyces*[6].

### Construction of the recombinant strains

The *rimP* disruption mutant (rimPDM) of *E. coli* BW25113 was constructed by PCR targeting as follows: A 1.2 kb DNA fragment containing the kanamycin resistance gene (*kan*) was amplified by PCR using primers ECrimP-F and ECrimP-R. This fragment covered the 38-bp upstream region and the 65-bp downstream region of *rimP*. Then the fragment was purified and introduced into the BW25113 by electroporation. Finally, the kanamycin resistance gene substituted the most of *rimP* coding region by homologous recombination. The resulting strain was confirmed by PCR amplification using primers YZECrimP-F and YZECrimP-R. In order to clarify the relationship between *rimP* of *E. coli* and SCO5703 of *S. coelicolor*, the heterologous complemented strain was constructed according to the following steps: Firstly, promoter region of *rrnF* was amplified with primer rrnFp-F and rrnFp-R from *S. coelicolor* and ligated into pEASY-blunt to generate pEASY-blunt-rrnFp. The authenticity of PCR amplicon was verified by sequencing, and then it was ligated into the *Not*I-*Bam*HI site of integrative vector pSET152 to give pSET152::rrnFp. Meanwhile, the DNA fragment containing the intact SCO5703 was amplified by PCR using primers PFrimP-F and PFrimP-R, then it was digested with *Xba*I-*Bam*HI and ligated into the corresponding sites of pSET152::rrnFp to generate pSET152::rrnFp::SCO5703. Finally, rimPDM was transformed with the plasmid pSET152::rrnFp::SCO5703 to generate the heterologous complemented strain of rimPDM (rimPDMC).

To construct the *rimP-SC* disruption mutant (rimP-SCDM) of *S. coelicolor* M145, the DNA fragment corresponding to the upstream region of *rimP-SC* (extending from positions −1269 to +12 with respect to the *rimP-SC* translation start codon) was amplified by PCR using primers LrimP-SC-F and LrimP-SC-R and inserted into the *Hin*dIII*-Xba*I sites of pUC119::*kan* to generate pRIMPSC1. The DNA fragment corresponding to the downstream region of *rimP-SC* (extending from positions +403 to +1576 with respect to the *rimP-SC* translation start codon) was amplified by PCR using primers RrimP-SC-F and RrimP-SC-R and inserted into the *Kpn*I*-Eco*RI sites of pRIMPSC1. The resulting plasmid pRIMPSC2 was then digested with *Hin*dIII and *Eco*RI. A 3.3 kb DNA fragment was isolated and ligated into the corresponding sites of pKC1132 to give pRIMPSC3. The authenticity of all PCR amplicons was verified by sequencing. Subsequently, pRIMPSC3 was introduced into *S. coelicolor* M145 via ET12567/pUZ8002 by conjugal transfer and the transformants conferring kanamycin resistance (Kan^r^) and apramycin sensitivity (Apr^s^) were selected*,* and they were further confirmed by PCR using primers LrimP-SC-F and RrimP-SC-R. For complementation analysis, the fragment containing the intact *rimP-SC* with its putative promoter region was amplified with primers CrimP-SC-F and CrimP-SC-R and inserted into the *Eco*RV site of pSET152 to generate pRIMPSC4. Subsequently, pRIMPSC4 was introduced into rimP-SCDM by conjugal transfer and the complemented strain was confirmed by PCR.

To construct the *rimP-SV* disruption mutant (rimP-SVDM) of *S. venezuelae* ATCC10712, the DNA fragment corresponding to the upstream region of *rimP-SV* was amplified by PCR using primers LrimP-SV-F and LrimP-SV-R and inserted into the *Hin*dIII*-Xba*I sites of pKC1139 to generate pRIMPSV1. The DNA fragment corresponding to the downstream region of *rimP-SV* was amplified by PCR using primers RrimP-SV-F and RrimP-SV-R and inserted into the *Bam*HI-*Eco*RV sites of pRIMPSV1 to generate pRIMPSV2. Kanamycin resistance gene was amplified by PCR using primers Kan-F and Kan-R and inserted into the *Bam*HI*-Xba*I sites of pRIMPSV2 to generate pRIMPSV3. The authenticity of all PCR amplicons was verified by sequencing. Subsequently, pRIMPSV3 was introduced into *S. venezuelae* ATCC10712 via ET12567/pUZ8002 by conjugal transfer and transformants conferring kanamycin resistance (Kan^r^) and apramycin sensitivity (Apr^s^) were selected, and they were further confirmed by PCR using primers YZrimP-SV-F and YZrimP-SV-R. For complementation analysis, the fragment containing the intact *rimP-SV* with its putative promoter region was amplified using primers CrimP-SV-F and CrimP-SV-R and inserted into the *Eco*RV site of pSET152 to generate pRIMPSV4. Subsequently, pRIMPSV4 was introduced into rimPSV-DM by conjugal transfer and the complemented strain was confirmed by PCR.

For detection of MetK and SigR expression in *S. coelicolor*, the 3 × FLAG-tagged system was applied and series of plasmids were constructed as follows: The DNA fragment containing the intact *metK* or *sigR* with its respective promoter was amplified by PCR with primers metK-F/metK-R or sigR-F/sigR-R and ligated into the *Stu*I site of pIJ10500 to generate pIJ10500::*metK* or pIJ10500::*sigR*. The resulting plasmid pIJ10500::*metK* or pIJ10500::*sigR* was introduced into *S. coelicolor* M145 and rimP-SCDM by conjugal transfer, respectively. All the recombinant strains were subsequently confirmed by PCR amplification.

### RNA isolation, RT-PCR and real-time RT-PCR

Total RNA were isolated from *Streptomyces* as described previously [36,37]. For reverse transcription PCR (RT-PCR) and quantitative real-time reverse transcription PCR (real-time RT-PCR), the genomic DNA was removed from RNA samples with RQ1 RNase-free DNase (Promega), the synthesis of the first-strand cDNA was performed with Superscript III first-strand Synthesis System (Invitrogen) as described previously [38]. Reaction mixtures contained 6 pmol of random primers (Invitrogen) and 1 μg of RNA in a total volume of 20 μl. The reverse transcription conditions were as follows: 65°C for 5 min, 25°C for 5 min, 50°C for 45 min, 55°C for 45 min, and 72°C for 10 min. RT-PCR reaction parameters were as follows: 95°C for 5 min, followed by 30 amplification cycles consisting of 95°C for 30 seconds denaturation, 55°C for 30 seconds annealing, 72°C for 45 seconds extension and a final extension of 72°C for 10 min. RT-PCR was performed without reverse transcriptase to test for DNA contamination in the RNA samples. After 30 cycles of amplification, the products were displayed on a 2% agarose gel and visualized by staining with ethidium bromide. Real-time RT-PCR was performed in 96-well rotor using the Eppendorf Realplex system, and the reaction mixtures were prepared as follows: Each reaction (50 μl) contained 0.1-10 ng of cDNA, 25 μl Power SYBR Green PCR master mix (Toyobo, QPS-201), and 0.4 μM of forward and reverse primers respectively. The reaction conditions were maintained at 95°C for 30 seconds, followed by 40 amplification cycles consisting of 15 seconds denaturation at 95°C, 20 seconds annealing at 60°C and 30 seconds extension at 72°C. Fluorescence was measured at the end of each cycle. The final dissociation stage was run to generate a melting curve and consequently verify the specificity of the amplification products. Changes in levels of gene expression were calculated automatically with the Detection Software using the ΔΔCT method. The *hrdB* was used as the housekeeping gene reference for RT-PCR and real-time RT-PCR.

### Construction of the *xylE* reporter system and Detection of translational fidelity

The *xylE* was isolated from pIJ4083 by *Bgl*II and *Bam*HI digestions, and then it was inserted into the *Bam*HI site of pSET152 to generate pSET152::*xylE*. The DNA fragment containing the *rrnF* promoter from pEASY-blunt-rrnFp was isolated and inserted in the upstream of *xylE* in pSET152 to generate pSET152::rrnFp::xylE. For the construction of mutated *xylE* reporter plasmid, pSET152::rrnFp::xylE was used as the template for PCR amplification with primers MxylE-F and MxylE-R. The authenticity of PCR amplicon was verified by sequencing. The mutated plasmid pSET152::rrnFp::xylE^*^, which contained alterations in the 5′ region of the *xylE* gene, was introduced a premature stop codon that abolished catechol dioxygenase activity. The reporter plasmids pSET152::rrnFp::xylE and pSET152::rrnFp::xylE^*^ had the correct orientation of promoter in favor of transcriptional detection of *xylE*, and then both of them were introduced into BW25113 and rimPDM to estimate translational fidelity. The translational error rate was calculated as activity of the wild-type catechol dioxygenase divided by that of mutated catechol dioxygenase in the same strain.

### Activity assays of XylE

The detailed steps for activity detection of catechol dioxygenase were performed as described previously with minor revised [6]. For the recovery of recombinant strains, they were inoculated in 3 ml of LB and incubated for 8–10 h at 37°C with shaking at 220 rpm. Then, the same amount of cells of each strain was transferred to 50 ml of LB medium and incubated for 3.5, 5.5, 7, 9, 12 or 23 h, respectively. Cultures of 1 ml were harvested. After washing with 1 ml sample buffer (100 mM phosphate buffer pH 7.5, 20 mM EDTA pH 8.0, 10% acetone), they were re-suspended in 0.5 ml of sample buffer. The 0.5 ml of cell suspension was sonicated on ice and 5 μl of 10% Triton X-100 was added. It was placed on ice for 15 min and centrifuged for 10 min at 12,000 rpm, the supernatants were used for activity assays of XylE. The reaction mixture for measurement of catechol dioxygenase activity consisted of 0.5 ml of assay buffer (10 mM phosphate buffer, pH 7.5, 0.2 mM catechol) and 5–50 μl of cell extract. Protein concentrations of cell extracts were measured according to the BCA protein assay method by using BSA as the standard. The catechol dioxygenase activity was calculated as the rate of change in optical density at 375 nm per minute per milligram of protein. The formula is as follows: catechol dioxygenase (mU) = 30.03 **×** △A_375_/time (min).

### Western blotting of MetK and SigR in *S. coelicolor*

For western blotting analysis, cell extracts from M145/pIJ10500::*metK*, M145/pIJ10500::*sigR*, rimP-SCDM/pIJ10500::*metK* and rimP-SCDM/pIJ10500:*:sigR*, grown in the GYM medium at different time points, were sonicated on ice. The concentration of total protein was determined by BCA protein assay using BSA as the standard sample. Equal concentrations of proteins (50 μg) from different time-point samples were loaded onto 12% polyacrylamide/SDS gel electrophoresis. Proteins in the gels were transferred to PVDF western blotting membranes (Roche, Germany) and probed with monoclonal ANTI-FLAG M2 antibody (Sigma-aldrich, USA) as recommended by the manufacturer. The antibodies on the membranes were hybridized with the goat Anti-Mouse IgG-HRP as secondary antibody (Jackson, USA) and the position of tagged-FLAG was visualized through cECL western blot kit (CWBIO Corporation, China).

### Heterologous expression and bioassays of polyoxin and nikkomycin

The entire polyoxin and nikkomycin biosynthetic gene clusters were ligated with integrated vector pSET152 to generate pPOL and pNIK, respectively [34,35]. Then they were introduced into M145 and rimP-SCDM to generate recombinant strains M145/pPOL, rimP-SCDM/pPOL, M145/pNIK and rimP-SCDM/pNIK. Recombinant strains containing the entire polyoxin or nikkomycin biosynthetic gene cluster were confirmed by PCR amplification using primers polB-F/polB-R or sanG-F/sanG-R respectively. Then 5 days’ fermentation broths of all the recombinant strains were measured by a disk agar diffusion method using *A. longipes* as indicator strain.

## Competing interests

The authors declare that they have no competing interests.

## Authors’ contributions

YP and CL performed the experiments of *S. coelicolor* and *S. venezuelae* respectively. HD carried out the HPLC analysis of ppGpp. LY assisted with experiments. YP wrote the draft manuscript. GL and HT supervised the whole work and revised the manuscript. All authors read and approved the final manuscript.
